# Harmonizing methods for wildlife abundance estimation and pathogen detection in Europe—a questionnaire survey on three selected host-pathogen combinations

**DOI:** 10.1186/s12917-016-0935-x

**Published:** 2017-02-16

**Authors:** Jana Sonnenburg, Marie-Pierre Ryser-Degiorgis, Thijs Kuiken, Ezio Ferroglio, Rainer G. Ulrich, Franz J. Conraths, Christian Gortázar, Christoph Staubach, APHAEA project partners, Pelayo Acevedo, Andreas Agreiter, Anna Bajer, Alex Barlow, Charalambos Billinis, Franck Boue, Andrea Cadamuro, Maria S. Calabrese, Gioia Capelli, Isabel Lopes de Carvalho, Adriano Casulli, Ermanno Cetto, Mario Chiari, Leo J. M. Dekkers, Peter Deplazes, J. Paul Duff, Javier Millán Gasca, Walter Glawischnig, Irina Golovljova, Roland Grunow, Jean Hars, Marja Isomursu, Jens Jacob, Kastriot Korro, Antonio Lavazza, Jane Learmount, Annick Linden, Andrzej Lipowski, Miriam Maas, Ignasi Marco, Roman Meier, Marcos Miñarro Prado, Viacheslav Morozov, Sofia Núncio, Riccardo Orusa, Thomas Romig, Sophie Rossi, Francisco Ruiz-Fons, Gianmaria Sommavilla, Michal Stanko, Adolf Steinrigl, Herbert Tomaso, Jurga Turcinaviciene, Umberto Zamboni

**Affiliations:** 1 Federal Research Institute for Animal Health, Friedrich-Loeffler-Institut, Institute of Epidemiology, Südufer 10, 17493 Greifswald, Insel Riems Germany; 2 National Veterinary Institute, Technical University of Denmark, Bülowsvej 27, Building 1-6, 1870 Frederiksberg C, Denmark; 3 Centre for Fish and Wildlife Health, Department Infectious Diseases and Pathobiology, Vetsuisse Faculty, University of Bern, Länggass-Str. 122, Postfach 8466, CH-3001 Bern, Switzerland; 4 Department of Viroscience, Erasmus Medical Centre, P.O. Box 2040, Ee1726, 3000 CA Rotterdam, The Netherlands; 5 Dipartimento di Scienze Veterinarie, Università degli Studi di Torino, Largo Paolo Braccini, 2 (già Via L. DaVinci, 44), 10095 Grugliasco, TO Italy; 6 Federal Research Institute for Animal Health, Institute of Novel and Emerging Infectious Diseases, Friedrich-Loeffler-Institut, Südufer 10, 17493 Greifswald, Insel Riems Germany; 7 SaBio Instituto de Investigaciónen Recursos Cinegéticos IREC Universidad de Castilla—La Mancha & CSIC, Ronda de Toledo s/n, 13071 Ciudad Real, Spain

**Keywords:** Animal abundance, Diagnostic methods, Europe, Harmonization, Questionnaire, Wildlife

## Abstract

**Background:**

The need for wildlife health surveillance as part of disease control in wildlife, domestic animals and humans on the global level is widely recognized. However, the objectives, methods and intensity of existing wildlife health surveillance programs vary greatly among European countries, resulting in a patchwork of data that are difficult to merge and compare. This survey aimed at evaluating the need and potential for data harmonization in wildlife health in Europe. The specific objective was to collect information on methods currently used to estimate host abundance and pathogen prevalence. Questionnaires were designed to gather detailed information for three host-pathogen combinations: (1) wild boar and Aujeszky’s disease virus, (2) red fox and *Echinococcus multilocularis*, and (3) common vole and *Francisella tularensis*.

**Results:**

We received a total of 70 responses from 19 European countries. Regarding host abundance, hunting bags are currently the most widely accessible data source for widely distributed mid-sized and larger mammals such as red fox and wild boar, but we observed large differences in hunting strategies among countries as well as among different regions within countries. For small rodents, trapping is the method of choice, but practical applications vary among study sites. Laboratory procedures are already largely harmonized but information on the sampled animals is not systematically collected.

**Conclusions:**

The answers revealed that a large amount of information is available for the selected host-pathogen pairs and that in theory methods are already largely harmonized. However, the comparability of the data remains strongly compromised by local differences in the way, the methods are applied in practice. While these issues may easily be overcome for prevalence estimation, there is an urgent need to develop tools for the routine collection of host abundance data in a harmonized way. Wildlife health experts are encouraged to apply the harmonized APHAEA protocols in epidemiological studies in wildlife and to increase cooperation.

**Electronic supplementary material:**

The online version of this article (doi:10.1186/s12917-016-0935-x) contains supplementary material, which is available to authorized users.

## Background

The need for wildlife health surveillance as part of disease control in wildlife, domestic animals and humans on the global level is now widely recognized. Increasing efforts are made to implement wildlife health surveillance schemes in various parts of the world. The Working Group on Wildlife of the World Organization for Animal Health (OIE) biannually collects, compiles and distributes information on a global scale. In Europe, the objectives, methods and intensity of existing wildlife health surveillance programs vary greatly among countries [1]. This results in a patchwork of data, which are difficult to merge and compare. Harmonized methods could allow a more reliable description of the disease status in populations on regional and continental scales. Efficient communication and coordination beyond national borders are required for effective early warning, because the migration of wildlife populations is usually not limited by borders between countries and globalization dramatically increases the risk of pathogen movements. Also, the understanding of factors influencing pathogen maintenance and spread in populations requires the application of similar investigation methods allowing reliable comparisons among regions with different patterns of pathogen occurrence [2].

The setup of a European wildlife health surveillance network under the umbrella of the European Wildlife Disease Association (EWDA [3]) in autumn 2009 represented an important first step to improve communication and data exchange among institutions involved in wildlife health surveillance in Europe [1]. In 2012, an EMIDA ERA-NET [4] project “harmonized Approaches in monitoring wildlife Population Health, and Ecology and Abundance” (acronym APHAEA, [5]) was started in close association with EWDA activities. The project aimed to develop a European wildlife disease surveillance network capable of providing reliable estimates on abundance of wildlife species and pathogen distribution and occurrence in key wildlife species in order to improve wildlife health surveillance in general. First, literature reviews on methods for estimating wildlife population abundance (leading to the production of ‘Species cards’) and for wildlife disease diagnostics (‘Diagnosis cards’) were conducted with the aim to propose selected methods for data harmonization on a large scale (APHAEA protocols [5]). The second step in the APHAEA project consisted in evaluating the willingness of wildlife health experts throughout Europe to participate in harmonization efforts and to contribute to case studies on host abundance and pathogen prevalence, using the three following host-pathogen combinations as examples: (1) wild boar (*Sus scrofa*) and Aujeszky’s disease virus (ADV; pseudorabies virus, *Suid herpesvirus 1*), (2) red fox (*Vulpes vulpes*) and *Echinococcus multilocularis* (*E. multilocularis*; alveolar echinococcosis), and (3) common vole (*Microtus arvalis*) and *Francisella tularensis* (*F. tularensis*; tularemia). Thus, the selection included an ungulate, a carnivore and a rodent as hosts as well as a virus, a parasite and a bacterium with economic or zoonotic importance as pathogens. Pathogens, for which diagnostic standards already exist by legislation of the European Union (EU), were deliberately excluded.

This article reports the results of a questionnaire survey performed among wildlife health experts who volunteered to participate in the APHAEA project. The survey aimed at describing the current situation and discussing the needs and potential of harmonizing methods in wildlife health research in Europe. More specifically, information was collected on: (1) the methods used to estimate host abundance and the availability of such data and (2) the methods used for pathogen detection and the availability of laboratory results together with sample metadata, for the three above-mentioned host-pathogen combinations in European countries.

## Methods

From summer 2012 to early 2013, wildlife health experts throughout Europe were informed about APHAEA by an oral presentation at the EWDA 2012 conference, written documents distributed through wildlife health internet forums and individual email contacts. The contacted experts were asked to forward the information to any potentially concerned colleagues in their respective countries. Altogether, 53 persons from 25 institutions in 18 European countries registered as external APHAEA project partners during that time period.

Questionnaires were designed to collect detailed information on the abundance of each selected host and on prevalence estimates for each corresponding pathogen. Information based on historical records was asked for as well as currently available data or information potentially accessible in the future. There was one questionnaire per host-pathogen combination [Additional files 1, 2 and 3].

The questionnaires included questions on the considered region (name, size), the existing data sources for animal abundance, the performed hunting schemes (for wild boar and fox), the temporal and regional scales, for which the data were available, and the recorded animal metadata. In addition, information was gathered on the occurrence of the selected pathogens or diseases in animal hosts (epidemic vs. endemic status or disease freedom) and in humans (for tularemia). Description of the geographical origin of samples was based on the Nomenclature of Territorial Units for Statistics (NUTS), a geocode standard for referencing subdivisions of countries for statistical purposes. There are three NUTS levels, with two more detailed levels of local administrative units (LAUs) [6]. Participants were also asked, whether data from former, on-going or future investigations were available.

APHAEA recommended the following protocols for wild animal abundance: hunting bag data on a large scale and more accurate density estimates (such as thermal imaging and distance sampling, camera-trapping or drive counts) on a local scale for wild boar; spot light counts for red fox; and snap trapping for common vole (APHAEA protocols [5]). For pathogen prevalence estimation the following diagnostic protocols were recommended by APHAEA: enzyme-linked immunosorbent assay (ELISA) for ADV; the sedimentation and counting technique (SCT) for *E. multilocularis*; and polymerase chain reaction (PCR) for *F. tularensis* (APHAEA protocols [5]).

The questionnaires were circulated among experts from the five APHAEA core partner countries (Spain, Italy, Switzerland, Germany and The Netherlands) and sent to all registered external partners in October 2013. All partners were invited to pass the questionnaires to other colleagues active in the wildlife field in their respective countries.

## Results

**Fig. 1:**
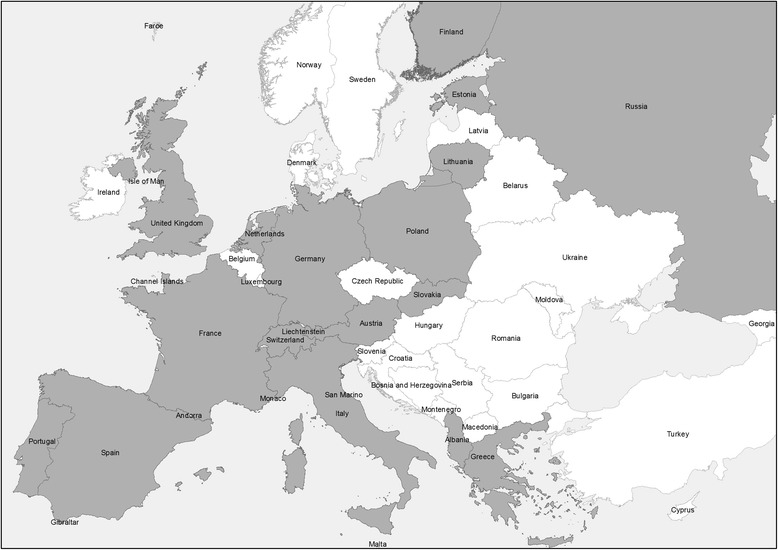
Map of Europe showing the countries for which a questionnaire was completed for at least one host-pathogen combination (*dark grey areas*)

We received a total of 70 completed questionnaires from 19 European countries (Fig. 1 and Acknowledgements). Responses were obtained on all three host-pathogen pairs from seven countries, on two host-pathogen pairs from five countries, and on one host-pathogen pair from six countries. Seventeen of 53 experts, who had registered as APHAEA partners (33%), answered at least to one questionnaire. In addition, 31 non-registered experts filled in questionnaires. A total response ratio could not be calculated because the number of experts who finally received the questionnaires was not known.

### Wild boar and Aujeszky’s disease virus

**Fig. 2:**
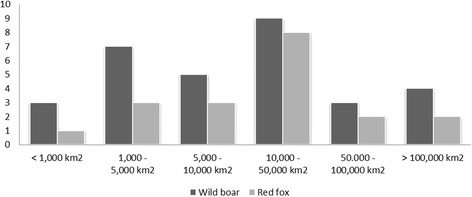
Number of study areas categorized by size of study area in km2 reported by the questionnaire respondents for wild boar and Aujeszky’s disease virus (*n* = 31) and for red fox and Echinococcus multilocularis (*n* = 18)

We received 31 completed questionnaires for the host-pathogen combination wild boar and ADV. The size of the considered study areas within countries ranged from 28 to 640,679 km^2^ (mean = 71,195 km^2^; median = 10,391 km^2^; Fig. 2).

#### Host abundance-related questions

**Fig. 3:**
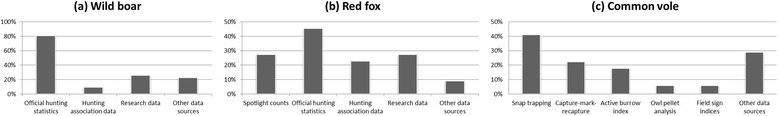
Existing data sources on the abundance of **a** wild boar (*n* = 31), **b** red foxes(*n* = 22) and **c** common voles (*n* = 17) as reported by questionnaire respondents (multiple answers were possible)

The reported data sources for wild boar abundance are shown in Fig. 3a. More accurate methods such as drive counts, snow tracking, kilometric abundance index, pellet counts, capture-mark-recapture and photo-trapping were applied by academic groups for research purposes. Other data sources included crop and car damage statistics, the wild boar surveillance database of the EU reference laboratory for Classical Swine Fever/African Swine Fever (EURL CSF/ASF-WB-DB, [7]), predictions obtained by modelling hunting bags and interviews of hunters, game wardens or veterinarians. For 16 study areas (52%), only hunting bag data were available. Nine respondents (29%) indicated to have access to both hunting statistics and to more accurate abundance data. For three study areas, only data generated by alternative methods were available.

**Fig. 4:**
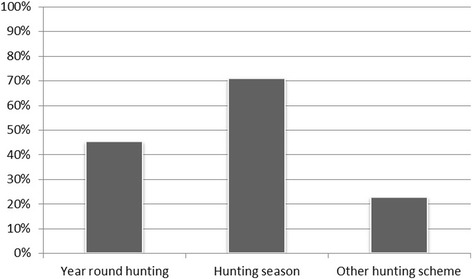
Wild boar hunting scheme for wild boar in the considered study areas as reported by questionnaire respondents (*n* = 31, multiple answers were possible). Other management schemes include e.g. selective culling by official game managers for population reduction

In most study areas, hunting was allowed in specific season(s) or performed year-round with seasonal peaks (Fig. 4). However, 11 different hunting seasons were mentioned. Hunting was allowed at least from October to December, but never in April in 21 of 22 study areas. In one questionnaire, it was reported that hunting was performed in the opposite year period, i.e. from February to September and not in the usual autumn/winter months. Other management schemes such as selective culling by official game managers for population reduction were reported for five study areas in addition to the regular hunting periods or as independent measures.

The regional and temporal scale as well as additional information from the accessible wild boar data is shown in Table 1. Overall, data accuracy and specifications varied considerably among countries and study areas.

**Table 1:** Regional scale, time scale and additional information from the accessible wild boar data as reported by questionnaire respondents

	Official hunting statistics (*n* = 25)	Hunting association data (*n* = 3)	Research data (*n* = 8)	Other*** (*n* = 7)
Regional scale				
NUTS 1*	10	1	0	1
NUTS 2*	13	2	1	1
NUTS 3*	17	2	2	0
LAU 1*	8	1	6	0
LAU 2*	12	2	5	1
Other (e.g. hunting grounds, hunting estates, 10 × 10 km)	10	0	1	1
Time scale				
Month	2	1	2	0
Quarter	0	1	1	0
Year	23	3	6	3
Other (e.g. hunting season, daily)	9	0	1	1
Additional information				
Age class	13	1	9	3
Weight	7	1	9	1
Sex	12	1	9	3
Cause of death**	12	2	7	2
Others (e.g. *Trichinella* testing, fecundity, general health)	0	0	0	1

* See [7] for more information

** E.g. found dead, shot sick, road traffic accident, regular hunting

*** e.g. EURL CSF/ASF-WB-DB

Four of the 31 respondents (15%) declared that hunting statistics were recorded in the EURL CSF/ASF-WB-DB for their countries.

#### Pathogen-related questions

**Fig. 5:**
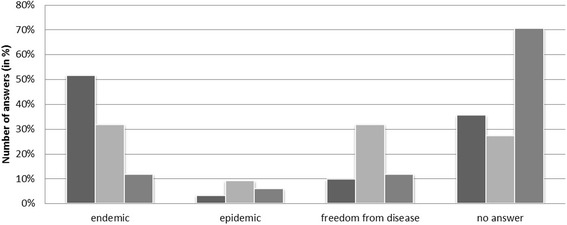
Disease status regarding Aujeszky’s disease in wild boar (*dark grey bars*, *n* = 31), *Echinococcus multilocularis* in red foxes (*light grey bars*, *n* = 22) and *Francisella tularensis* in common voles (*middle grey bars*, *n* = 17) in the considered study areas as reported by questionnaire respondents

The status of ADV in wild boar as reported by the survey participants is presented in Fig. 5. The source of information was stated in 14 cases and consisted mostly of research data (71%), but also of information available from official ADV surveillance (partly in domestic pigs) or from annual monitoring programs in wild boar (29%). Diagnostic findings of Aujeszky’s disease in hunting dogs and information from veterinary officials were mentioned as an additional source of information for one study area.

Seventeen partners (55%) stated that historical data were available, in 21 study areas (68%) investigations were on-going at the time of the survey and in three study areas (10%) investigations were planned. A total lack of information and absence of studies was only reported for one (3%) of the study areas. Table 2 summarizes the information that was or may become available for wild boar samples from these investigations on ADV.

**Table 2:** Information available for wild boar samples from ongoing, historical or planned investigations on Aujeszky’s disease virus as reported by questionnaire respondents

	Ongoing (*n* = 21)	Historical (*n* = 17)	Planned (*n* = 3)
Age class	15	12	3
Sex	15	12	3
Date	15	11	3
Location	16	13	3
Cause of death ^a^	10	4	2
Results of serological investigations	16	12	3
Results of genetic and virological investigations	6	7	3

^a^ E.g. found dead, shot sick, road traffic accident, regular hunting

Eighteen laboratories participating in the survey stated that they were able to investigate wild boar samples serologically and 12 were capable of detecting the virus or its genome (four by PCR, two by virus isolation and one by immunofluorescence assay). Laboratories without a possibility to test samples in their own country stated that they were willing to send serum samples (12 partners) or tissue samples (five partners) to a foreign laboratory.

### Red fox and *E. multilocularis*

We received 22 completed questionnaires for the host-pathogen combination red fox and *E. multilocularis*. The size of the considered study areas ranged from 865 to 239,000 km^2^ (mean = 45,290 km^2^, median = 23,861 km^2^, Fig. 2).

#### Host abundance-related questions

The sources of information on red fox abundance are shown in Fig. 3b. These data sources were even more heterogeneous than for wild boar, but hunting statistics were also the main data source for fox abundance. Furthermore, hunting statistics regarding foxes were available for longer time periods (up to 80 years, median = 15 years) than data generated by other methods (up to 9 years, median = 3 years).

Ten study areas (45%) practice year-round hunting with seasonal peaks (*n* = 3) and a protection period for females with pups (*n* = 1). For seven study areas, the hunting season was indicated to last from September until January/February (32%), while hunting took place in the opposite season, i.e. from July to February, in one study area (5%).

Table 3 show the regional and temporal scale, at which information on red fox abundance was available for the considered study areas. Like for wild boar, the available metadata are very inconsistent among countries and areas.

**Table 3:** Regional scale, time scale and additional information available from the red fox data as reported by questionnaire respondents

	Spotlight counts (*n* = 6)	Official hunting statistics (*n* = 10)	Hunting association data (*n* = 5)	Research data (*n* = 6)
Regional scale				
NUTS 1 ^a^	0	2	0	0
NUTS 2 ^a^	1	3	1	1
NUTS 3 ^a^	1	4	1	2
LAU 1 ^a^	1	0	1	2
LAU 2 ^a^	1	0	2	1
Other (e.g. experimental field area)	5	4	3	2
Time scale				
Month	1	0	1	2
Quarter	1	0	1	1
Year	6	11	5	1
Other not specified by the participants)	1	0	0	3
Additional information				
Age class	0	3	1	4
Sex	0	4	1	4
Cause of death ^b^	0	7	4	3

^a^See [7] for more information

^b^Type of carcass, e.g. found dead, shot sick, road traffic accident, regular hunting

Only eleven partners (50%) mentioned that they could record data by spotlight counting in the framework of future studies.

#### Pathogen-related questions

The status of the occurrence of *E. multilocularis* reported by the survey participants is shown in Fig. 5.

Numerous studies on *E. multilocularis* in red fox have been performed, are on-going or planned, with very variable sample sizes. Eight survey participants (36%) stated that they were able to investigate samples by the intestinal scraping technique in their own laboratory. Seven laboratories (32%) could perform SCT, 13 laboratories offered PCR (59%) and 4 laboratories applied other techniques (18%), including EmsB microsatellite analysis (*n* = 3) and a copro-antigen ELISA (*n* = 1) One participant did not specify the applied methods. Seven participants (32%) confirmed that they were able to send sera and tissue samples (intestines, intestinal mucosa, carcasses, feces or isolated specimens of *E. multilocularis*) to another laboratory, while seven participants (32%) denied that they could do so.

Table 4 presents the potentially available information on samples from ongoing, historical or planned studies.

**Table 4:** Information available for samples from ongoing, historical or planned investigations on *Echinococcus multilocularis* as reported by questionnaire respondents

	Ongoing (*n* = 8)	Historical/finished (*n* = 11)	Planned (*n* = 5)
Age class^a^	7	7	3
Sex	8	7	3
Collection date	8	9	3
Location	8	9	3
Cause of death^b^	3	5	2
Results of intestinal scraping technique	2	5	0
Results of sedimentation and counting technique	2	4	0
Results of PCR	4	6	2
Results of other investigations	1	4	1
Other^c^	1	2	0

^a^Age categorization, e.g. juvenile, adult

^b^Type of carcass, e.g. found dead, shot sick, road traffic accident, regular hunting

^c^additional intestinal parasites, reproduction status, body condition and size estimates

### Common vole and *F. tularensis*

We received 17 completed questionnaires for the host-pathogen combination common vole and *F. tularensis*.

#### Host abundance-related questions

The source of information on common vole abundance is shown in Fig. 3c. Again, data sources were heterogeneous. The snap trapping method (recommended APHAEA protocol) is currently applied by only 7% of the respondents (2–20 plots). Nevertheless, abundance data from snap trapping were available for 6 to 72 years (median = 20 years). Active burrow index data and data from owl pellet analyses were available for 20 or 6 years, respectively, while field sign indices were available for only 1 to 2 years.

Seven participants (41%) stated that it would be possible to survey populations by snap trapping for common voles following the APHAEA protocol, whereas four participants (24%) denied due to animal welfare issues.

#### Pathogen-related questions

Tularemia was mentioned as a notifiable disease in 14 of the participating countries (82%). Of these 14 countries, 10 (59%) reported human cases of tularemia in 2012, whereas three (18%) did not register any human cases and four (24%) failed to provide the relevant information. The reported tularemia status of *F. tularensis* in the considered study areas is shown in Fig. 5.

Few studies were performed in the past, five were on-going and more were planned at the time of the survey. Five participants (29%) indicated to have the possibility to investigate samples in their own laboratory by culture methods and other tests (isolation and typing, special nutrient media resistance, biological test with the subsequent growth of material on special media). Nine partners (53%) could perform PCR in their laboratories and six (35%) use serological techniques like the microagglutination test, ELISA, Western blot or the immunofluorescence antibody assay. Ten partners (59%) with access to common vole samples, but without possibility to test them for *F. tularensis*, were able to send tissue samples to another laboratory, and eight respondents (47%) could send serum samples.

In four study areas (24% of the respondents), data on *F. tularensis* were available for other rodents belonging to the families Cricetidae, and Muridae.

The available information on common vole samples from ongoing and permanent, historical or planned studies is shown in Table 5. Host information was collected only in the framework of snap trapping and included species (6 of 7 studies), age class (4 of 7 studies) and sex (6 of 7 studies).

**Table 5:** Available information on common vole samples in ongoing, historical or planned studies as reported by questionnaire respondents

	Ongoing (*n* = 5)	Historical (*n* = 8)	Planned (*n* = 6)
Age class	3	4	4
Sex	3	4	4
Date	3	4	5
Results of culture methods and tests ^a^	1	1	2
Results of PCR	1	2	5
Results of serological investigations ^a^	1	1	2

^a^Specified as IFA in one case

## Discussion

Effective wildlife disease surveillance and investigations of disease dynamics in wildlife populations requires knowledge of wildlife population sizes, their dynamics and changes in the geographical distribution over time. Such information is required to design appropriate sampling protocols for pathogen/disease surveys, to develop disease contingency plans, to assess the risk of pathogen transmission among different species and to guide wildlife management strategies in general [8]. However, for coordinated health surveillance efforts on a large scale, methods for assessing host population abundance and for detecting pathogen occurrence need to be harmonized in order to obtain comparable data [9].

The aim of this questionnaire survey was to clarify the potential for harmonizing methods in wildlife health research and population monitoring on the continental scale in Europe. Attempts to describe host abundance or disease/pathogen occurrence across countries were previously performed using available data, but they focused mostly on the host or the pathogen, on pooled data without information on their heterogeneity or underlined the difficulty to perform reliable comparisons [10–14]. To our knowledge, this article is the first to report the heterogeneity of the existing data in detail and the resulting limitations concerning their use for meta-studies. This is illustrated by the types of data available for three pairs of key host species and disease agents.

Wildlife experts showed a strong willingness to participate in the APHAEA project in general and in the questionnaire survey in particular. The participation of only a few European countries might have led to an incomplete and biased picture of the situation in Europe. Scandinavian and numerous Eastern European countries had registered as APHAEA partners and participated in networking activities concerning wildlife health, although they were not represented in the survey (Fig. 1). Scandinavia is known to carry out general wildlife health surveillance [1] and there are published data on *F. tularensis* and *E. multilocularis* in wildlife from Scandinavia and Eastern Europe [15, 16]. Therefore, the non-participation of countries in this specific survey does not necessarily reflect a lack of interest or a lack of activity.

However, our study included 68% of the EU member states and the three host-pathogen combinations referred to very different hosts and pathogens. The results obtained for each combination highlighted various challenges regarding the harmonization of methods for estimating host abundance or detecting pathogen occurrence. Some of these challenges were common for most or all studied regions, others were specific for individual areas or countries. Other host-pathogen combinations might have shown different levels of harmonization end even more difficulties; nevertheless, we believe that our results provide in principle an adequate overview on already performed harmonization efforts and the problematic lack of harmonization in other areas.

The present questionnaire survey revealed hunting bags are currently the most widely accessible data source that or hunted mid-sized and large mammals such as red fox and wild boar. While hunting bags may generate interesting data on a large scale [10, 14], their use remains controversial, because they are not readily comparable among countries and even among study areas within the same country due to different hunting efforts and hunting regulations [14]. However, more accurate methods for wildlife abundance estimation, i.e. those generating animal density estimations rather than hunting-based estimations, are time-consuming and costly. This makes it more difficult to applied them on a large scale and on the long term [17–20]. Therefore, the few data at the moment, available on live animal densities need to be combined with alternative data sources including hunting bags to model wildlife host abundances. In parallel, improved collaboration between experts in wildlife health and in wildlife ecology is required to develop tools for host abundance estimations which fulfill the need of epidemiological investigations.

For small mammals, which are not hunted, such as common voles, methods for abundance estimations vary widely, which poses even more severe challenges for comparing study areas. Due to the absence of relatively easily accessible data such as hunting bags, damage statistics and traffic accidents, which can deliver helpful information on population trends when combined, harmonization efforts are even more urgent to enable work at the continental scale.

By contrast, methods for pathogen detection are largely harmonized for all three considered pathogens due to a limited number of available techniques. Furthermore, EU regulations already exist for the selection of diagnostic methods for numerous pathogens and represent invaluable support towards harmonized procedures. Nevertheless, the collection of metadata on the samples is not yet sufficiently systematic. Information on the animals, from which the samples originate, is essential for data interpretation and comparisons among regions and countries [21]. It is therefore important to strive for a better harmonization of diagnostic data beyond laboratory procedures in the future.

The proposed APHAEA protocols have the potential to contribute to a more comprehensive harmonization on a European scale. Nevertheless, their application is not mandatory and implementing new methods require human and financial resources, which are usually limited. Overall, harmonization needs a common will to strive to a common goal, whether it concerns host abundance or pathogen detection, research or monitoring. We ultimately need to build a baseline for European-wide co-operations in disease surveillance and control, where wildlife management plays an essential role. The EURL CSF/ASF-WB DB is a good example on how data on wild boar hunting bags and disease surveillance could be standardized and combined on large scale.

## Conclusions

A solid basis for a harmonized wildlife disease surveillance scheme exists in Europe, which integrates information on host populations and pathogens. The situation regarding the harmonization of methods for pathogen diagnosis is encouraging, but there are still opportunities for improvement. Importantly, much work needs to be done regarding the harmonization of wildlife abundance estimation methods. Therefore, we urge wildlife health experts to consider the recommended methods for harmonization for both pathogen detection and species abundance estimation in future studies (so-called APHAEA protocols, www.aphaea.org or www.ewda.org) to collect metadata on the animals concerned and to collaborate in the development of practice-oriented tools for wildlife abundance estimations.

## Additional files

Additional file 1: Questionnaire on wild boar and Aujeszky’s disease virus. (PDF 912 kb)
Additional file 2: Questionnaire on red fox and *Echinococcus multilocularis*. (PDF 1009 kb)
Additional file 3: Questionnaire on common vole and *Francisella tularensis*. (PDF 2040 kb)

## Abbreviations

ADV: Aujeszky’s disease virus
APHAEA: harmonized Approaches in monitoring wildlife Population Health, and Ecology and Abundance
*E. multilocularis*: *Echinococcus multilocularis*
ELISA: Enzyme-linked immunosorbent assay
EURL CSF/ASF-WB-DB: European Union reference laboratory Classical Swine Fever/African Swine Fever wild boar surveillance database
EWDA: European Wildlife Disease Association
*F. tularensis*: *Francisella tularensis*
IST: Intestinal scraping technique
LAU: Local administrative unit
NUTS: Nomenclature of Territorial Units for Statistics
PCR: Polymerase chain reaction
SCT: Sedimentation and counting technique
