# Supplementary material for: Harmonizing methods for wildlife abundance estimation and pathogen detection in Europe—a questionnaire survey on three selected host-pathogen combinations
Source: BMC Vet Res. 2017 Feb 16;13:53. doi: 10.1186/s12917-016-0935-x (PMC5312528; doi:10.1186/s12917-016-0935-x)
Supplement: Additional file 1: — Questionnaire on wild boar and Aujeszky’s disease virus. (PDF 912 kb) [file 12917_2016_935_MOESM1_ESM.pdf]

Wild boar & Aujeszky's disease virus
Questionnaire on population data and samples

Guidelines for data usage

This questionnaire is designed to collect information regarding historical records, data currently available or potentially accessible in the future. After potential co-operation partners have been identified on basis of the answers in the questionnaire, we will provide further information, protocols and Excel-sheets to facilitate data exchange.

Any data you provide to the APHAEA project will be treated as strictly confidential and will only be used within the framework of the project for the selection of feasible studies for the evaluation of harmonized sampling protocols. It is planned to publish the harmonization efforts, strengths and maybe occurred problems of the protocols based on the evaluation of the provided data. The manuscript will be send to the data providers prior to publication and your co-authorship will be recognized. In any case, it is planned to share the results of the questionnaire evaluation in an aggregated, anonymous form among the participants of the survey.

If there are any questions, please do not hesitate to contact us for further information via feedback@aphaea.eu.

Content

Guidelines for data usage.....1
Content.....1
Personal information.....2
Wild boar (*Sus scrofa*) & Aujeszky's disease virus.....2
Appendix.....7

Personal information

1.1 Country:

1.2 Full name:

1.3 Organization:

1.4 Email:

1.5 If it is not yet the case, would you be willing to have your name / organization listed in the external partners' list on the APHAEA website (www.aphaea.org)?

Name: ○ yes ○ no

Organization: ○ yes ○ no

Wild boar & Aujeszky's disease virus

Population related questions

2.1 Please describe **the region considered for the study.** If there is more than one region considered, please fill the questionnaire several times.

Name of region:

Size (in sqkm):

Comment:

2.2 Which data sources exist in your country providing information on the wild boar density (multiple choices are possible)**?**

- ☐ Official hunting statistics (collected through official sources)
- ☐ Hunting association data (collected through private associations)
- ☐ Research data (collected by academic groups) e.g. capture-mark-re-capture, pellet
- ☐ Other:

2.3 What kind of **hunting strategy / scheme** is performed in the considered region? Please fill in text for explanation.

- ☐ Year-round (e.g. constant, seasonal peaks)
- ☐ Hunting season (from month to month)
- ☐ Other:

2.4 Is the wild boar density information marked in 2.2 available **for the region considered in 2.1** for at least 5 years **(2012 and previous years)?**

	Official hunting statistics	Hunting association data	Research data	Other
Yes				
No				

2.5 On which **regional scale** is the wild boar density information available for the region considered in 2.1 (please see table 1 in the Appendix section for more details)?

	Official hunting statistics	Hunting association data	Research data	Other
NUTS 1				
NUTS 2				
NUTS 3				
LAU 1				
LAU 2				
Other: e.g. hunting grounds, GPS				

2.6 On which **time scale** is the wild boar density information available for the region considered in 2.1?

	Official hunting statistics	Hunting association data	Research data	Other
Month				
Quarter				
Year				
Other:				

2.7 Which **additional information** is collected in the wild boar density information?

	Official hunting statistics	Hunting association data	Research data	Other
Age class (see Appendix)	☐	☐	☐	☐
Weight	☐	☐	☐	☐
Sex	☐	☐	☐	☐
Type of carcass (see Appendix)	☐	☐	☐	☐
Other:	☐	☐	☐	☐

2.8 Are your hunting statistics of wild boar recorded in the EU Reference Laboratory **„Classical swine fever in wild boar surveillance database“** (http://public.csf-wildboar.eu)?

○ yes ○ no

Would it be possible to get the **permission to use data** of the hunting bag from this database?

○ yes ○ no

Disease related questions

All questions refer to **Aujeszky's disease in wild boar** and the region mentioned in 2.1. If there are disease related data only for a sub region of the considered area, please specify the size of the sub region in sqkm:

2.9 Did or does **Aujeszky's disease in wild boar** occur within the region considered above?

○ Endemic infection ○ Epidemic infection ○ Freedom from disease

☐ Historical data available ☐ Ongoing actual data ☐ No investigations / studies conducted in the area

What is the source of your information? e.g. official hunting statistics, research data

2.10 Could data from former, ongoing or future **investigations about Aujeszky's disease virus in wild boar** from the region (or a sub region) mentioned in 2.1 be available for the APHAEA project?

Ongoing	◯ yes	◯ no	from	to
Finished	◯ yes	◯ no	from	to
Permanent	◯ yes	◯ no	since	
Planned	◯ yes	◯ no	from	to

2.11 Please fill in the **number of collected samples** that could be used within the APHAEA project referring to the investigations mentioned in 2.10.

	Ongoing	Finished	Permanent	Planned
Sample size for serological investigations				
Sample size for PCR				

2.12 If there are planned investigations of Aujeszky's disease virus, **would you be able to investigate samples** at your laboratory?

☐ **Serologically** ☐ **Virologically** by e.g. PCR or virus isolation

2.13 If there are historical, ongoing, permanent or planned wild boar sample collections in your country but you do not have the possibility to test the samples for Aujeszky's disease virus, would it be possible **to send sera and/or tissue samples to another laboratory**?

Sera samples: ◯ yes ◯ no

Tissue samples: ◯ yes ◯ no

2.14 Would you have the possibility to **provide historical laboratory test results** of a former investigation regarding Aujeszky's disease virus in wild boar from the considered region?

◯ yes ◯ no

2.15 If there are samples (ongoing, historical or planned for future), which **information** is / will be available?

	Ongoing	Historical	Planned
Age class	☐	☐	☐
Sex	☐	☐	☐
Date	☐	☐	☐
Location<br>on which regional scale (see 2.5 and Appendix)	☐	☐	☐
Carcass (see Appendix)	☐	☐	☐
Results of **serological investigations** (if performed)	☐	☐	☐
Results of **virological investigations** (if performed)	☐	☐	☐
Other:	☐	☐	☐

General questions

2.16 Please list any **publications concerning wild boar population data and Aujeszky's disease** within the considered region and time.

2.17 Additional comments:

Appendix

Age class

Age categorisation, e.g. < 1 year, 1-2 years, > 2 years

Carcass

Type of carcass, e.g. found dead, shot sick, road traffic accident, regular hunting

NUTS classification

Table 1 The NUTS classification (Nomenclature of territorial units for statistics) as hierarchical system for dividing up the economic territory of the EU (source: http://epp.eurostat.ec.europa.eu/portal/page/portal/ nuts_nomenclature/correspondence_tables/national_s tructures_eu)

Country (abbr.)	NUTS 1	NUTS 2	NUTS 3	LAU 1	LAU 2
**BE**	Gewesten / Régions	Provinces / Provinces	Arrondissementen / Arrondissements	-	Gemeenten / Communes
**BG**	Райони (Rajoni)	Райони за планиране (Rajoni za planirane)	Области (Oblasti)	Общини (Obshtini)	Населени места (Naseleni mesta)
**CZ**	Území	Oblasti	Kraje	Okresy	Obce
**DK**	-	Regioner	Landsdeler	Kommuner	Sogne
**DE**	Länder	Regierungsbezirke	Kreise	Verwaltungs-gemeinschaften	Gemeinden
**EE**	-	-	Groups of Maakond	Maakond	Vald, linn
**IE**	-	Regions	Regional Authority Regions	Counties, Cities	Electoral Districts
**GR**	Γεωγραφική Ομάδα (Groups of development regions)	Περιφέρειες (Periferies)	Νομοί (Nomoi)	Δήμοι, Κοινότητες (Demoi, Koinotites)	Δημοτικά Διαμερίσματα, Κοινοτικά Διαμερίσματα (Demotiko diamerisma, Koinotiko diamerisma)
**ES**	Agrupacion de comunidades Autonomas	Comunidades y ciudades Autonomas	Provincias + islas + Ceuta, Melilla	-	Municipios
**FR**	Z.E.A.T + DOM	Régions + DOM	Départements + DOM	Cantons de rattachement	Communes
**IT**	Gruppi di regioni	Regioni	Provincie	-	Comuni
**CY**	-	-	-	Επαρχίες (Eparchies)	Δήμοι, Κοινότητες (Dimoi, koinotites)
**LV**	-	-	Statistiskie reģioni	-	Republikas pilsētas, novadi
**LT**	-	-	Apskritys	Savivaldybės	Seniūnijos
**LU**	-	-	-	Cantons	Communes
**HU**	Statisztikai nagyrégiók	Tervezési-statisztikai régiók	Megyék + Budapest	Statisztikai kistérségek	Települések
**MT**	-	-	Gzejjer	Distretti	Kunsilli
**NL**	Landsdelen	Provincies	COROP regio's	-	Gemeenten
**AT**	Gruppen von Bundesländern	Bundesländer	Gruppen von politischen Bezirken	-	Gemeinden

Country (abbr.)	NUTS 1	NUTS 2	NUTS 3	LAU 1	LAU 2
**PT**	Continente + Regioes autonomas	Comissaoes de Coordenaçao regional + Regioes autonomas	Grupos de Concelhos	Concelhos - Munícipios	Freguesias
**RO**	Macroregiuni	Regiuni	Judet + Bucuresti	-	Comuni + Municipiu + Orase
**SI**	-	Kohezijske regije	Statistične regije	Upravne enote	Občine
**SK**	-	Oblasti	Kraje	Okresy	Obce
**FI**	Manner-Suomi, Ahvenananmaa / Fasta Finland, Åland	Suuralueet / Storområden	Maakunnat / Landskap	Seutukunnat / Ekonomiska regioner	Kunnat / Kommuner
**SE**	Grupper av riksområden	Riksområden	Län	-	Kommuner
**UK**	Government OHce Regions; Country	Counties (some grouped); Inner and Outer London; Groups of unitary authorities	Upper tier authorities or groups of lower tier authorities (unitary authorities or districts)	Lower tier authorities (districts) or individual unitary authorities; Individual unitary authorities or LECs (or parts thereof); Districts	Wards (or parts thereof)
