# Supplementary material for: Harmonizing methods for wildlife abundance estimation and pathogen detection in Europe—a questionnaire survey on three selected host-pathogen combinations
Source: BMC Vet Res. 2017 Feb 16;13:53. doi: 10.1186/s12917-016-0935-x (PMC5312528; doi:10.1186/s12917-016-0935-x)
Supplement: Additional file 2: — Questionnaire on red fox and Echinococcus multilocularis. (PDF 1009 kb) [file 12917_2016_935_MOESM2_ESM.pdf]

Red fox & *Echinococcus multilocularis*
Questionnaire on population data and samples

Guidelines for data usage

This questionnaire is designed to collect information regarding historical records, data currently available or potentially accessible in the future. After potential co-operation partners have been identified on basis of the answers in the questionnaire, we will provide further information, protocols and Excel-sheets to facilitate data exchange.

Any data you provide to the APHAEA project will be treated as strictly confidential and will only be used within the framework of the project for the selection of feasible studies for the evaluation of harmonized sampling protocols. It is planned to publish the harmonization efforts, strengths and maybe occurred problems of the protocols based on the evaluation of the provided data. The manuscript will be send to the data providers prior to publication and your co-authorship will be recognized. In any case, it is planned to share the results of the questionnaire evaluation in an aggregated, anonymous form among the participants of the survey.

If there are any questions, please do not hesitate to contact us for further information via feedback@aphaea.eu.

Content

Guidelines for data usage....1
Content....1
Personal information....2
Red fox (*Vulpes vulpes*) & *Echinococcus multilocularis*....2
Appendix....8

Personal information

1.1 Country:

1.2 Full name:

1.3 Organization:

1.4 Email:

1.5 If it is not yet the case, would you be willing to have your name / organization listed in the external partners' list on the APHAEA website (www.aphaea.org)?

Name: ◯ yes ◯ no

Organization: ◯ yes ◯ no

Red fox & *Echinococcus multilocularis*

Population related questions

2.1 Please describe **the region considered for the study.** If there is more than one region considered, please fill the questionnaire several times.

Name of the region:

Size (in sqkm):

Comment:

2.2 Which data sources exist in your country providing information on the red fox density (multiple choices are possible)**?**

- ☐ Spotlight counts
- ☐ Official hunting statistics (collected through official sources)
- ☐ Hunting association data (collected through private associations)
- ☐ Research data (collected by academic groups) e.g. capture-mark-re-capture
- ☐ Other:

2.3 What kind of **hunting strategy / scheme** is performed in the considered region? Please fill in text for explanation.

- ☐ Year-round (e.g. constant, seasonal peaks)
- ☐ Hunting season (from month to month)
- ☐ Other:

2.4 For which **time period** is the fox density information marked in 2.2 **available** for the region considered in 2.1 (2012 and previous years)?

	Spotlight counts	Official hunting statistics	Hunting association data	Research data	Other
Time period					

2.5 On which **regional scale** is the population density information available for the region considered in 2.1 (please see table 1 in the Appendix section for more details)?

	Spotlight counts	Official hunting statistics	Hunting association data	Research data	Other
NUTS 1	☐	☐	☐	☐	☐
NUTS 2	☐	☐	☐	☐	☐
NUTS 3	☐	☐	☐	☐	☐
LAU 1	☐	☐	☐	☐	☐
LAU 2	☐	☐	☐	☐	☐
Other: e.g. hunting grounds, GPS, (sub-) region	☐	☐	☐	☐	☐

2.6 On which **time scale** is the red fox population density information available for the region considered in 2.1?

	Spotlight counts	Official hunting statistics	Hunting association data	Research data	Other
Month	☐	☐	☐	☐	☐
Quarter	☐	☐	☐	☐	☐
Year	☐	☐	☐	☐	☐
Other: e.g. spring and autumn	☐	☐	☐	☐	☐

2.7 Which **additional information** is collected in the hunting statistics on the red fox population?

	Official hunting statistics	Hunting association data	Research data	Other
Age class (see Appendix)	☐	☐	☐	☐
Sex	☐	☐	☐	☐
Type of carcass (see Appendix)	☐	☐	☐	☐
Other:	☐	☐	☐	☐

2.8 Would it be possible to record data regarding the fox population by spotlight counting?

○ yes ○ no

Disease related questions

All questions refer to ***Echinococcus multilocularis* in red foxes** and the region mentioned in 2.1. If there are disease related data only for a sub or supra region of the considered area, please specify the size of the sub or supra region (in sqkm):

2.9 Did or does *Echinococcus multilocularis* occur within the region considered above?

○ Endemic infection	○ Epidemic infection	○ Freedom from disease
☐ Historical data available	☐ Ongoing actual data	☐ No investigations / studies conducted in the area

What is the source of your information?

2.10 Could data from former, ongoing or future **investigations about *Echinococcus multilocularis* in red foxes** from the (sub or supra) region mentioned in 2.1 be available for the APHAEA project?

Ongoing	○ yes	○ no	from	to
Finished	○ yes	○ no	from	to
Permanent	○ yes	○ no	since	
Planned	○ yes	○ no	from	to

2.11 Please fill in the **number of collected samples** that could be used within the APHAEA project referring to the investigations mentioned in 2.10.

**Sample size for**	Ongoing	Finished	Permanent	Planned
Intestinal scraping technique				
Sedimentation and counting technique				
PCR				
Other techniques				

2.12 If there are planned investigations of *Echinococcus multilocularis* in red foxes, **would you be able to investigate samples** at your laboratory?

- ☐ Intestinal scraping technique
- ☐ Sedimentation and counting technique
- ☐ PCR (Please specify, e.g. real-time PCR)
- ☐ Other:

2.13 If there are historical, ongoing, permanent or planned red fox sample collections in your country but you do not have the possibility to test the samples for *Echinococcus multilocularis*, would it be possible **to send sera and/or tissue samples** (intestines, intestinal mucosa, carcasses, feces or *Echinococcus multilocularis*) **to another laboratory**?

Sera samples: ○ yes ○ no

Tissue samples: ○ yes ○ no

2.14 Would you have the possibility to **provide historical laboratory test results** of a former investigation regarding *Echinococcus multilocularis* in red foxes within the considered region?

○ yes ○ no

2.15 If there are samples (ongoing, historical or planned for future), which **information** is / will be available?

	Ongoing	Historical	Planned
Age class	☐	☐	☐
Sex	☐	☐	☐
Collection date	☐	☐	☐
Location on on which regional scale (see 2.5 and Appendix)	☐	☐	☐
Carcass (see Appendix)	☐	☐	☐
Results of **investigations** (Intestinal scraping technique)	☐	☐	☐
Results of **investigations** (Sedimentation and counting technique)	☐	☐	☐
Results of **investigations** (PCR)	☐	☐	☐
Results of **investigations** (Other: method)	☐	☐	☐
Other:	☐	☐	☐

General questions

2.16 Please list any **publications concerning red fox population data and *Echinoccocus multilocularis*** within the considered region and time.

2.17 Additional comments:

Appendix

Age class

Age categorisation, e.g. juvenile, adult

Carcass

Type of carcass, e.g. found dead, shot sick, road traffic accident, regular hunting

NUTS classification

Table 1 The NUTS classification (Nomenclature of territorial units for statistics) as hierarchical system for dividing up the economic territory of the EU (source: http://epp.eurostat.ec.europa.eu/portal/page/portal/ nuts_nomenclature/correspondence_tables/national_s tructures_eu)

Country (abbr.)	NUTS 1	NUTS 2	NUTS 3	LAU 1	LAU 2
**BE**	Gewesten / Régions	Provincies / Provinces	Arrondissementen / Arrondissements	-	Gemeenten / Communes
**BG**	Райони (Rajoni)	Райони за планиране (Rajoni za planirane)	Области (Oblasti)	Общини (Obshtini)	Населени места (Naseleni mesta)
**CZ**	Území	Oblasti	Kraje	Okresy	Obce
**DK**	-	Regioner	Landsdeler	Kommuner	Sogne
**DE**	Länder	Regierungsbezirke	Kreise	Verwaltungs-gemeinschaften	Gemeinden
**EE**	-	-	Groups of Maakond	Maakond	Vald, linn
**IE**	-	Regions	Regional Authority Regions	Counties, Cities	Electoral Districts
**GR**	Γεωγραφική Ομάδα (Groups of development regions)	Περιφέρειες (Periferies)	Νομοί (Nomoi)	Δήμοι, Κοινότητες (Demoi, Koinotites)	Δημοτικά Διαμερίσματα, Κοινοτικά Διαμερίσματα (Demotiko diamerisma, Koinotiko diamerisma)
**ES**	Agrupacion de comunidades Autonomas	Comunidades y ciudades Autonomas	Provincias + islas + Ceuta, Melilla	-	Municipios
**FR**	Z.E.A.T + DOM	Régions + DOM	Départements + DOM	Cantons de rattachement	Communes
**IT**	Gruppi di regioni	Regioni	Provincie	-	Comuni
**CY**	-	-	-	Επαρχίες (Eparchies)	Δήμοι, Κοινότητες (Dimoi, koinotites)
**LV**	-	-	Statistiskie reģioni	-	Republikas pilsētas, novadi
**LT**	-	-	Apskritys	Savivaldybės	Seniūnijos
**LU**	-	-	-	Cantons	Communes
**HU**	Statisztikai nagyrégiók	Tervezési-statisztikai régiók	Megyék + Budapest	Statisztikai kistérségek	Települések
**MT**	-	-	Gzejjer	Distretti	Kunsilli
**NL**	Landsdelen	Provincies	COROP regio's	-	Gemeenten
**AT**	Gruppen von Bundesländern	Bundesländer	Gruppen von politischen Bezirken	-	Gemeinden

**Country (abbr.)**	NUTS 1	NUTS 2	NUTS 3	LAU 1	LAU 2
**PT**	Continente + Regioes autonomas	Comissaoes de Coordenaçao regional + Regioes autonomas	Grupos de Concelhos	Concelhos - Munícipios	Freguesias
**RO**	Macroregiuni	Regiuni	Judet + Bucuresti	-	Comuni + Municipiu + Orase
**SI**	-	Kohezijske regije	Statistične regije	Upravne enote	Občine
**SK**	-	Oblasti	Kraje	Okresy	Obce
**FI**	Manner-Suomi, Ahvenananmaa / Fasta Finland, Åland	Suuralueet / Storområden	Maakunnat / Landskap	Seutukunnat / Ekonomiska regioner	Kunnat / Kommuner
**SE**	Grupper av riksområden	Riksområden	Län	-	Kommuner
**UK**	Government OHce Regions; Country	Counties (some grouped); Inner and Outer London; Groups of unitary authorities	Upper tier authorities or groups of lower tier authorities (unitary authorities or districts)	Lower tier authorities (districts) or individual unitary authorities; Individual unitary authorities or LECs (or parts thereof); Districts	Wards (or parts thereof)
