# Supplementary material for: Harmonizing methods for wildlife abundance estimation and pathogen detection in Europe—a questionnaire survey on three selected host-pathogen combinations
Source: BMC Vet Res. 2017 Feb 16;13:53. doi: 10.1186/s12917-016-0935-x (PMC5312528; doi:10.1186/s12917-016-0935-x)
Supplement: Additional file 3: — Questionnaire on common vole and Francisella tularensis. (PDF 2040 kb) [file 12917_2016_935_MOESM3_ESM.pdf]

Common vole & *Francisella tularensis*

Questionnaire on population data and samples

Guidelines for data usage

This questionnaire is designed to collect information regarding historical records, data currently available or potentially accessible in the future. After potential co-operation partners have been identified on basis of the answers in the questionnaire, we will provide further information, protocols and Excel-sheets to facilitate data exchange.

Any data you provide to the APHAEA project will be treated as strictly confidential and will only be used within the framework of the project for the selection of feasible studies for the evaluation of harmonized sampling protocols. It is planned to publish the harmonization efforts, strengths and maybe occurred problems of the protocols based on the evaluation of the provided data. The manuscript will be send to the data providers prior to publication and your co-authorship will be recognized. In any case, it is planned to share the results of the questionnaire evaluation in an aggregated, anonymous form among the participants of the survey.

If there are any questions, please do not hesitate to contact us for further information via feedback@aphaea.eu.

Content

Guidelines for data usage....1
Content....1
Personal information....2
Common vole (*Microtus arvalis*) & *Francisella tularensis*....2
Appendix 7

Personal information

1.1 Country:

1.2 Full name:

1.3 Organization:

1.4 Email:

1.5 If it is not yet the case, would you be willing to have your name / organization listed in the external partners' list on the APHAEA website (www.aphaea.org)?

Name: ○ yes ○ no

Organization: ○ yes ○ no

Common vole & *Francisella tularensis*

Population related questions

2.1 Please describe **the region considered for the study.** If there is more than one region considered, please fill the questionnaire several times.

Name of region:

Comment:

2.2 Which data sources exist in the considered region providing information on the common vole (or related rodents/hares) density/abundance (multiple choices are possible)?

- ☐ Snap trapping, following the snap trapping protocol: (see Appendix) ○ yes ○ no
- ☐ Capture-mark-recapture
- ☐ Active burrow index
- ☐ Owl pellet analysis
- ☐ Field sign indices: e.g. fresh soil, grass clippings
- ☐ Other: e.g. hair tubes

2.3 For which **time period** is the density information marked in 2.2 **available** for the region considered in 2.1 (2012 and previous years)?

	Snap trapping	Capture-mark-recapture	Active burrow index	Owl pellet analysis	Field sign indices	Other
Time period						

2.4 Which **additional information** is collected?

	Snap trapping	Capture-mark-recapture	Other
Species	☐	☐	☐
Age class	☐	☐	☐
Sex	☐	☐	☐
Other:	☐	☐	☐

2.5 If snap trapping is performed, how many **plots** were used for trapping within the considered region?

2.6 Is it possible to survey populations following the snap trapping standard protocol for *Microtus* (*arvalis)*?

○ yes ○ no

Disease related questions

All questions refer to ***Francisella tularensis* in common voles** (and related rodents or lagomorphs) and the region mentioned in 2.1. If there are disease related data only for a sub or supra region of the considered area, please specify the size of the sub or supra region (in sqkm):

2.7 Is *Francisella tularensis* a notifiable disease within your country?

○ yes ○ no

2.8 How many human cases of *Francisella tularensis* were reported?

e.g. number of cases during a specified time period

2.9 Did or does *Francisella tularensis* occur within the region considered above?

◯ Endemic infection	◯ Epidemic infection	◯ Freedom from disease
☐ Historical data available	☐ Ongoing actual data	☐ No investigations / studies conducted in the area

What is the source of your information?

2.10 Could data from former, ongoing or future **investigations about *Francisella tularensis* in common voles** (or related rodents) from the (sub or supra) region mentioned in 2.1 be available for the APHAEA project?

Ongoing	◯ yes	◯ no	from	year	to	year
Finished	◯ yes	◯ no	from	year	to	year
Permanent	◯ yes	◯ no	since	year		
Planned	◯ yes	◯ no	from	year	to	year

2.11 Please fill in the **number of collected samples** that could be used within the APHAEA project referring to the investigations mentioned in 2.10.

**Sample size for**	Ongoing	Finished	Permanent	Planned
Culture methods and tests				
PCR				
Serological investigations				
Other techniques				

2.12 If there are ongoing, permanent or planned investigations of *Francisella tularensis* in common voles (or related rodents), **would it be possible to investigate samples** at your laboratory?

☐ Culture methods and tests (e.g. biochemical investigations, cefinase test, erythromycin)

☐ PCR

☐ Serological investigations (e.g. SSA)

☐ Other:

2.13 If there are historical, ongoing, permanent or planned common vole (or related rodents) sample collections in your country but you do not have the possibility to test the samples for *Francisella tularensis*, would it be possible **to send tissue samples** (liver, spleen, lung, tonsils and/or kidney) **and/or sera to another laboratory**?

Tissue samples: ◯ yes ◯ no type of tissue

Sera samples: ◯ yes ◯ no

2.14 Would you have the possibility to **provide historical laboratory test results** of a former investigation regarding *Francisella tularensis* in common voles (or related rodents) within the considered region?

◯ yes ◯ no

If yes, was the trapping performed according to the snap trapping protocol (see Appendix)?

◯ yes ◯ no

If the trapping was **not** performed according to the snap trapping protocol, please describe the used trapping protocol:

2.15 Are disease related data regarding other rodents (Cricetidae, Muridae), that are additionally trapped, available?

○ yes ○ no

2.16 If there are samples (ongoing, historical or planned for future), which **information** is / will be available?

	Ongoing	Historical	Planned
Age class	☐	☐	☐
Sex	☐	☐	☐
Date	☐	☐	☐
Results of **culture methods and test** (method)	☐	☐	☐
Results of **PCR**	☐	☐	☐
Results of **serological investigations** (method)	☐	☐	☐
Results of other investigations (method)	☐	☐	☐
Other:	☐	☐	☐

General questions

2.17 Please list any **publications concerning common vole (or related rodents) population data and Francisella tularensis in the considered region and time.**

2.18 Additional comments:

Appendix

Snap trapping standard protocol for *Microtus* (*arvalis*)

Authors: **Stephan Drewes, Sabrina Schmidt, Rainer G. Ulrich (Friedrich-Loeffler-Institut, Greifswald-Insel Riems, Germany), Christian Imholt and Jens Jacob (Julius Kühn-Institute, Münster, Germany)**

1) Required permissions

- permissions (animal ethics, nature conservation etc.) have to be obtained prior to trapping according to national regulation
  - trapping has to be coordinated with land owner and user

2) *Microtus* abundance index estimation in grassland

- at least 3 plots on grassland are needed:
  - as far as possible grassland of each plot should be identical regarding composition, vegetation height and cover
  - on each plot no management actions are allowed for a period of 6 months prior to trapping
  - make sure that the land owner and land user are informed
  - no farming practice on plots shortly before and during trapping

3) Required materials

- metal or plastic snap traps (calculate 5 replacement traps per site)
- bait: raisins
- FFP3 respirator face mask
- protective gloves
- alcohol-based disinfective agent, e.g. mikrozid® AF liquid
- plastic (zipper) bags
- styrofoam package
- water-resistant marker pen
- appropriate clothes
- marker sticks or warning tape for flagging plots/ traps

4) Method

- distance between plots and the edge of the grassland to avoid edge effects: at least 20 m
- installation of 7 x 7 snap traps (60 x 60 m) baited with raisins in each plot (Figure 1)
- distance between single snap traps: 10 m
- mark the trap position using colored marker sticks or warning tape to find them again
- check snap traps once every 24 hours
- reset, replace released or missing traps and raisins

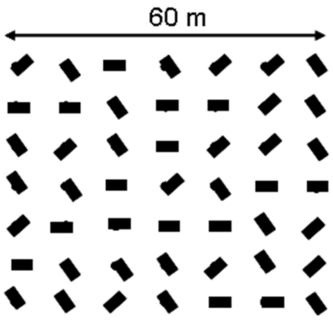

Figure 1 Installation scheme of 7x7 traps

5) **Calculation of the abundance index (trap success)**

- conduct trapping for at least of 100 trap nights
    - 1 trap night is defined by either 1 triggered trap with a captured *Microtus* vole or 1 not triggered trap with the bait still in place present for 24 h
    - triggered traps without bait and catch, or with non-target species caught as well as untriggered traps without bait are considered a failure, they need to be subtracted from the number of trap nights
- depending on the number of failures the trapping will at least take 3 nights until 100 trap nights are achieved
- trap success is the number trapped target individuals divided by the number of trap nights averaged across plots

6) **Handling, storage and transport of voles**

- morphological species determination if possible
- each vole should be immediately put into a labeled plastic (zipper) bag which should be closed
- label should provide information on: trapping site and corresponding plot, trapping date, trapped rodent species name and the name of the person doing the trapping
- plastic zipper bags from the same plot might be put together in a larger bag (label with site, date and the name of the person doing the trapping)
- the bags with the trapped animals should be frozen at -20°C as soon as possible
- to keep the animals frozen during the transport the styrofoam package must contain a sufficient amount of dry ice

The snap trapping standard protocol can also be found on

http://www.aphaea.org/sites/default/files/external/aphaea-workshop-standard-protocol-snap-trapping.pdf

You have to be registered as external partner on the website. Please contact Marie-Pierre.Ryser@vetsuisse.unibe.ch (please indicate “APHAEA” in the email title).
